# Usual or unusual presentations of *Dirofilaria repens* in two sibling dogs: a case report

**DOI:** 10.1007/s00436-020-06926-7

**Published:** 2020-10-20

**Authors:** Giulia Barlozzari, Tiziana Felice, Laura Salvato, Raffaella Conti, Claudio De Liberato, Federica Furzi, Simona Gabrielli, Manuela Scarpulla

**Affiliations:** 1 Istituto Zooprofilattico Sperimentale del Lazio e della Toscana M. Aleandri, Rome, Italy; 2 Public Health Unit RM3, Rome, Italy; 3 Department of Public Health and Infectious Diseases, Sapienza University of Rome, Rome, Italy

**Keywords:** *Dirofilaria repens*, Testicle, Circulating eosinophilia, Acute moist dermatitis, Dog, Italy

## Abstract

This study describes two different manifestations of *Dirofilaria repens* infection in sibling dogs with microfilaremia. Dog 1, asymptomatic, harbored a gravid female of *D. repens* on the parietal layer of *tunica vaginalis* of one testicle and showed a marked circulating eosinophilia (3.3·10^3^/μL). Both testicles were normal in shape and size without any gross lesions. Dog 2 had a pyotraumatic dermatitis. The cases were confirmed by PCR and sequencing. The sequences obtained showed 100% identity with those of *D. repens* isolated from human scrotum in Croatia. The treatment with moxidectin 2.5% and imidacloprid 10%/kg was effective in eliminating microfilariae after just one application, as demonstrated by negative modified Knott’s tests and PCR analyses of blood samples. This status was maintained during the post-treatment observation period. The classical localization of *D. repens* in dogs is in subcutaneous tissues, within nodules or free; however, it can also occur with some frequency in testicles, as described in humans. The infection can be associated with circulating eosinophilia or pyotraumatic dermatitis, as reported in this study. Thus, in endemic areas, it is advisable to carefully inspect the removed testicles at neutering since parasite localization can take place without any macroscopic changes. Moreover, in the case of circulating eosinophilia or pyotraumatic dermatitis, investigations should include modified Knott’s test and PCR to ensure that *D. repens* is not the cause of these alterations. Rapid and sensitive tests for the early detection of infected animals would help to prevent or limit the spread of this zoonosis.

## Introduction

The nematode *Dirofilaria repens* is responsible for canine subcutaneous dirofilariasis and it is the main agent of human dirofilariasis in the Old World. *D. repens* adult worms live in the subcutaneous and intramuscular connective tissues of dogs and other carnivores. The females, as viviparous, release the first larval stage (L1) in the peripheral blood after mating. The L1 is picked up by a mosquito while sucking blood, it turns into the second larval stage (L2) and finally into the infective stage (L3) that migrates towards the proboscis to be transmitted to a new host. The infective stage can be transmitted among dogs or to humans through the bite of several mosquito genera (*Aedes*, *Culex*, *Anopheles*, *Coquillettidia*). While there is a rapid test for the dog heartworm *Dirofilaria immitis*, no similar tests are available to detect *D. repens*–infected animals, making the undiagnosed dogs the main source of infection for other dogs and humans (Capelli et al. 2018).

In dogs, adult worms are usually localized in subcutaneous tissues, within nodules or free, and the infection is frequently subclinical. Clinical syndromes typically associated with *D. repens* infection in dogs are multifocal nodular dermatitis, generally localized to the face, and prurigo papularis dermatitis (Simón et al. 2012). Dermatological signs—such as pruritus, erythema, papules, focal/multifocal alopecia, hyperkeratosis, crusting, nodules, acanthosis, eczema, pyoderma, and edema—may appear and also recur seasonally in association with *D. repens* infection (Hargis et al. 1999, Tarello 2002, Tarello 2011, Rocconi et al. 2012, Albanese et al. 2013, Giudice et al. 2014). Some unusual localizations of the adult stage of *D. repens* in pelvic cavity, mesentery, bulbar conjunctival mass, and testicle have been reported (Hermosilla et al. 2006, Demiaszkiewicz et al. 2013, Ravindran et al. 2016, Mircean et al. 2017, Agapito et al. 2018, Omeragić et al. 2018, Napoli et al. 2019). In this report, we describe two different manifestations of *D. repens* infection in sibling dogs with microfilaremia. Dog 1, asymptomatic, harbored a gravid female of *D. repens* in a testicle and showed marked circulating eosinophilia, while dog 2 had an acute moist dermatitis without hematochemical changes: both cases were confirmed by polymerase chain reaction (PCR) and sequencing. Usual and unusual presentations of *D. repens* in dogs are also discussed in this paper.

## Materials and methods

On December 2018, two sibling Maremmano sheep dogs were admitted to a municipal kennel (“Muratella”, Rome, Italy) after their shepherd’s death. The dogs were 5–6 years old, male, around 40 kg and lived in the area of Fiumicino (N 41.86593, E 12.24963), near to Rome, where they worked as sheep-guarding dogs. At kennel admission, the dogs were treated with broad-spectrum ecto- and endo-parasiticide drugs: imidacloprid/permethrin (Advantix®, Bayer S.p.A.) and febantel/pyrantel pamoate/praziquantel (Drontal plus®, Bayer S.p.A.). Two months later, before neutering, as established by the Italian law for stray animals (DL 281/91), both dogs were clinically examined and submitted to a rapid test for the detection of specific antibodies against *D. immitis*, *Anaplasma phagocytophilum*, *Anaplasma platys*, *Borrelia burgdorferi*, *Ehrlichia canis*, and *Ehrlichia ewingii* (SNAP 4Dx Plus, Idexx). During the surgery, performed following the “closed” technique, a worm was found on the testicle surface of dog 1. The worm was sent to the “Istituto Zooprofilattico Sperimentale del Lazio e della Toscana” to be classified at species level, by both morphological and molecular analyses (Manfredi et al. 2001; Casiraghi et al. 2001). Additionally, an echocardiogram was performed on both dogs in order to exclude the presence of *D. immitis*, whereas a Baermann test was carried out only on dog 1 in order to exclude the presence of the lungworm *Angiostrongylus vasorum,* common cause of eosinophilia in dogs. Blood samples were collected into K3EDTA and serum tubes to be submitted for a complete cell blood count (CBC) (Abbott Cell-Dyn 3700, Abbott, IL, USA), blood chemistry (Olympus AU 400 Olympus, Tokyo, Japan), and serum electrophoresis. The presence of canine anti-*Leishmania* IgG antibodies in sera was checked using an in-house immunofluorescent-antibody test (IFAT). An enzyme-linked immunosorbent assay (ELISA) (PetChek Canine Heartworm Antigen Test, Idexx) was used to exclude the presence of circulating antigens of *D. immitis* adult female worms in sera. The presence of circulating microfilariae was assessed using a modified Knott’s test (Rawlings 1986). The molecular identification of the parasite was carried out in the DNA extracted from both the worm and whole blood samples using a commercial kit (QIAamp DNA Mini Kit, Qiagen). The extracted DNA was submitted to a PCR targeting a 650-bp fragment from the filariod-*cox*1 gene using the primers COIintF (5′-TGATTGGTGGTTTTGGTAA-3′) and COIintR (5′-ATAAGTACGAGTATCAATATC-3′) as described by Casiraghi et al. (2001), with slight modifications. DNA extracted from *D. repens* specimens, previously isolated and sequenced by Fontanelli Sulekova et al. (2016), and double-distilled water were included in PCR runs as positive and negative controls respectively. The PCR final products were separated by electrophoresis on a 1.5% agarose gel stained with Midori Green Advance DNA Stain (Nippon Genetics Europe GmbH, Düren, Germany) and examined under UV transilluminator. The amplicons of expected size were then purified and sequenced bi-directionally according to BigDye 1.1 technology using ABI3500 capillary sequencer (Applied Biosystems, Carlsbad, CA, USA). The resulting chromatograms were analyzed and edited using Geneious software (Biomatters Ltd.). The sequences obtained were compared to those previously deposited in GenBank by using the nBLAST algorithm (https://blast.ncbi.nlm.nih.gov/Blast.cgi).

## Results

### Clinical and laboratory results

**Fig. 1:**
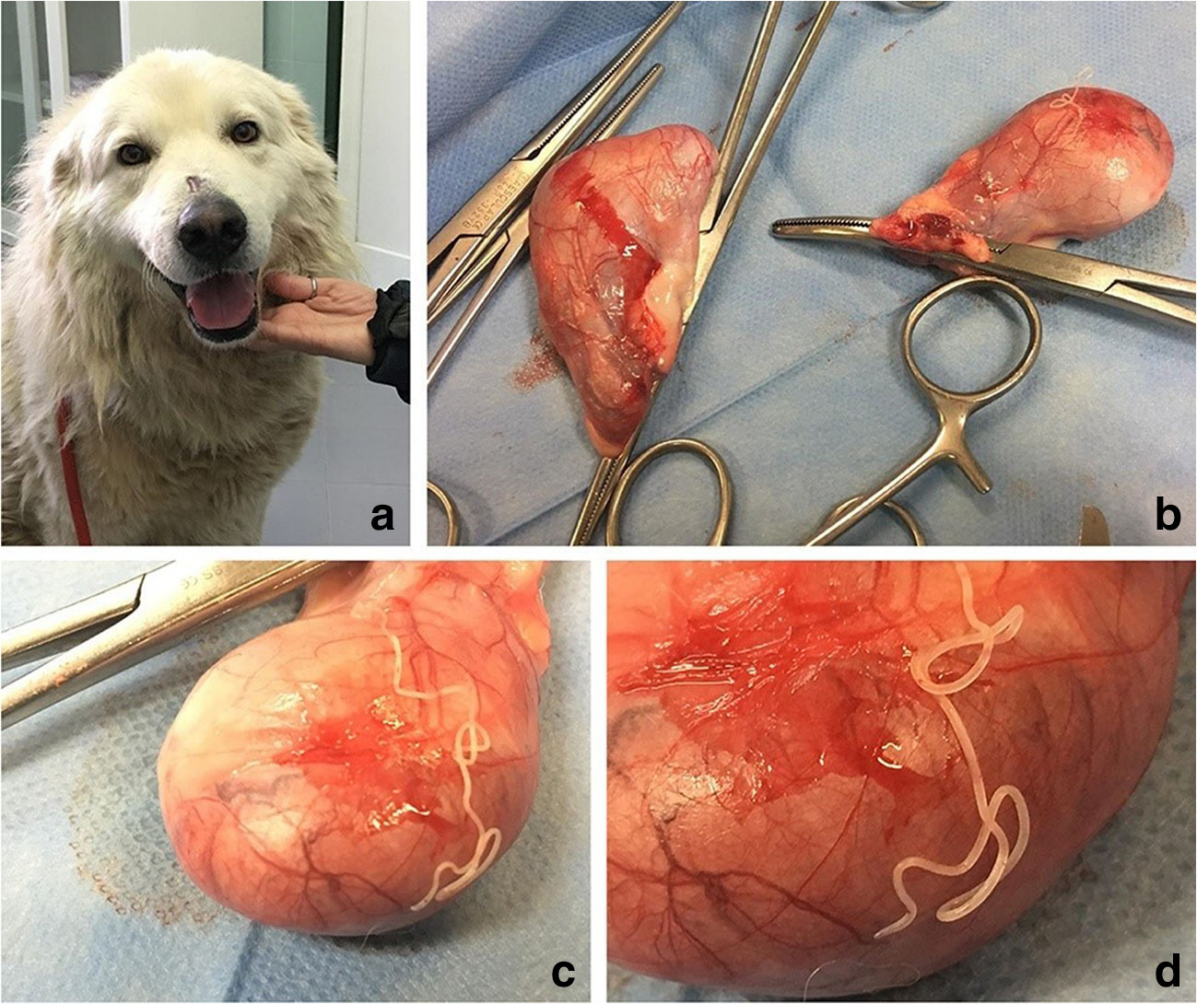
A: Dog 1; B–D: the testicles appear normal in shape and size without any gross lesions. A gravid female of *D. repens* is present on the surface of the parietal layer of *tunica vaginalis* of one testicle

**Fig. 2:**
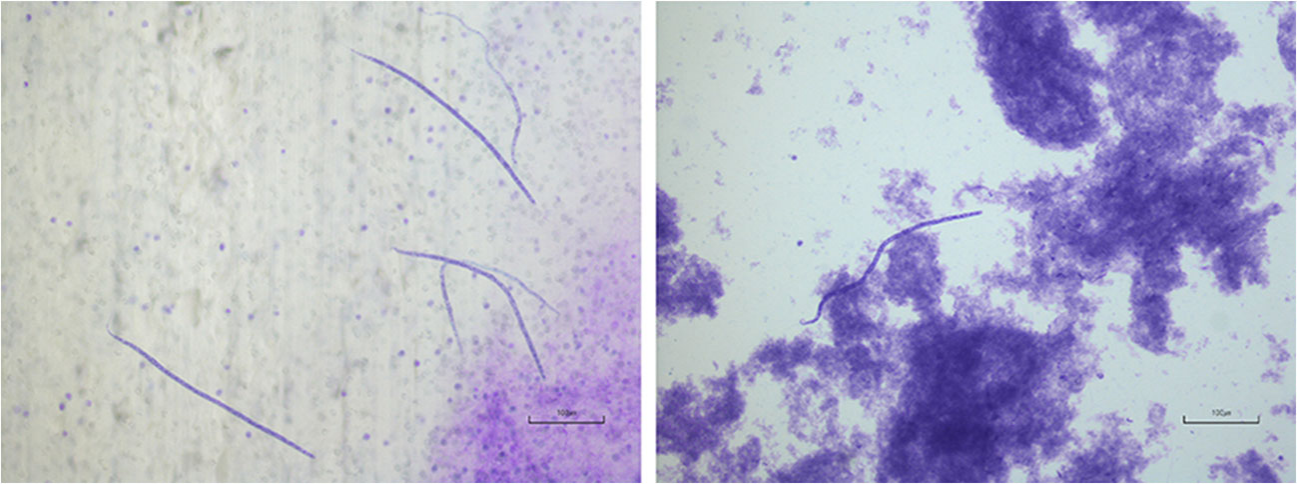
Modified Knott’s test: *D. repens* microfilariae from the dogs of this study

Dog 1: the worm found on the parietal layer of *tunica vaginalis* of one testicle was microscopically identified as a gravid female of *D. repens*. Both testicles were normal in shape and size without any gross lesions (Fig. 1). The dog showed circulating microfilariae (Fig. 2) with an estimated load of 302 microfilariae per milliliter. No abnormalities were found on physical examination or blood chemistry, while the CBC revealed a marked circulating eosinophilia (3.3·10^3^/μL). Serological analyses and Baermann’s test were negative. The echocardiogram was negative for heartworms. Dog 2: the dog was positive for circulating microfilariae (Fig. 2) with an estimated load of 800 microfilariae per milliliter. An acute moist dermatitis (wet eczema, hot spots, pyotraumatic dermatitis) was observed on the flank. Hematochemical profile was within the reference ranges and all serological tests turned out to be negative. The echocardiogram was negative for heartworms. Clinical and laboratory parameters of dogs are summarized in Table 1*.*

**Table 1:** Clinical and laboratory parameters of dogs infected by *D. repens* at first presentation

Clinical and laboratory parameters	Dog 1	Dog 2	Reference values
General physical examination	Normal	Acute moist dermatitis^a^	
Complete blood count			
RBC (10^6^/μL)	5.99	6.14	5.50–8.50
HGB (g/dL)	15.3	15	12.0–18.0
HCT (%)	41.4	41.6	37.0–55.0
MCV (fL)	68.9	67.8	60.6–77.0
MCH (pg)	25.5	24.4	19.5–24.5
MCHC (g/dL)	37.0	36.0	32.0–36.0
RDW (% CV)	16.6	17.2	12.0–18.0
WBC (10^3^/μL)	10.6	12.8	6.00–14.5
NEU (10^3^/μL)	5.34	8.14	3.00–11.0
LYM (10^3^/μL)	1.46	2.75	1.00–4.80
MONO (10^3^/μL)	0.446	0.79	0.1–1.30
EOS (10^3^/μL)	3.30^a^	1.0	0.1-1.2
BASO (10^3^/μL)	0.38	0.11	3.00–11.0
PLT (10^3^/μL)	183	163	200–500
Smear estimate of PLT numbers	Adequate	Adequate	
Serum chemistry			
ALT (U/L)	21	15	< 50
AST (U/L)	34	21	< 40
BUN (mg/dl)	13	18	8–23
CRE mg/dl)	1.02	0.96	(< 1.4
PT (g/dl)	5.9	5.8	5.4–7.1
Serum protein electrophoresis			
A/G (g/dl)	0.85	0.75	0.7–1.1
Alb (g/dl)	2.7	2.4	2.3–3.4
Alpha 1 (g/dl)	0.2	0.2	0.3–0.8
Alpha 2 (g/dl)	0.8	1.0	0.5–1.3
Beta 1 (g/dl)	0.7	0.8	0.3–0.8
Beta 2 (g/dl)	0.6	0.6	0.4–1.0
Gamma (0.4–1.0 g/dl)	0.9	0.8	
Other tests			
Modified Knott’s test (microfilariae/ml)	Pos (302)^a^	Pos (800)^a^	
PCR for *D. repens*	Pos	Pos	
*D. immitis* Antigen (ELISA)	Neg	Neg	
*L. infantum* IgG (IFAT)	Neg	Neg	
*E. canis* IgG (IFAT)	Neg	Neg	
*Anaplasma phagocytophilum*, *Anaplasma platys*, *Borrelia burgdorferi*, *Ehrlichia canis*, *Ehrlichia ewingii* (Snap Test)	Neg	Neg	
Baermann’s test	Neg	-	

^a^Altered parameters

### PCR and sequencing

**Fig. 3:**
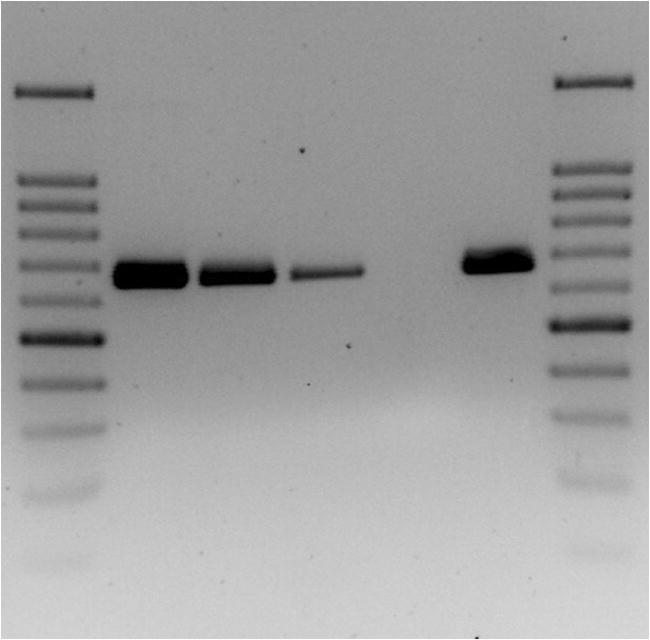
Filarioid *cox*1 PCR. Lane 1 and 7: 100-bp ladder; lane 2: dog 1 worm; lane 3: dog 1 blood; lane 4: dog 2 blood; lane 5: negative control; lane 6: *D. repens* positive control

Filarioid *cox-1* PCR gave positive results in the worm and blood samples of both dogs (Fig. 3). The sequences obtained (deposited under the accession numbers MT345574–MT345576) were100% identical to each other and showed 100% identity and 100% query cover with those of *D. repens*, accession number KX265049, isolated from human scrotum in Croatia.

### Therapy and follow-up

The dogs were monthly treated with moxidectin 2.5% and imidacloprid 10%/kg (Advocate® spot-on, Bayer S.p.A.) for the microfilaremia. The efficacy of the therapy was assessed monthly by modified Knott’s test and PCR in whole blood samples. Successfully, both tests turned out to be negative after the first treatment and remained negative during three monthly follow-ups. This status was maintained in dog 1 and dog 2 during a post-treatment observation period of 5 and 3 months respectively (Table 2). A CBC performed on dog 1 5 months after the last treatment showed a number of eosinophils of 0.9 × 10^3^/μL which was within the normal range (Table 1).

**Table 2:** Therapy and follow-up of microfilaremic dogs

	Control (*n*)	Treatment with moxidectin 2.5% and imidacloprid 10%/kg	Date (yyyy/mm/dd)	Modified Knott’s test (microfilariae/ml)	PCR for *D. repens*
Dog 1	1		2019/02/20	Pos (302)	Pos
	2	x	2019/04/11	Pos (800)	Pos
	3	x	2019/05/07	Neg	Neg
	4	x	2019/06/10	Neg	Neg
	5	x	2019/07/20	Neg	Neg
	6		2019/12/24	Neg	Neg
Dog 2	1		2019/02/20	Pos (800)	Pos
	2	x	2019/04/11	Pos (726)	Pos
	3	x	2019/05/07	Neg	Neg
	4	x	2019/06/13	Neg	Neg
	5	x	2019/07/20	Neg	Neg
	6		2019/10/11	Neg	Neg

## Discussion

In recent years, the number of reports focusing on uncommon localizations of adult stages of *D. repens* in dog is increasing. In particular, the testicular localization is not so unusual in dogs, as cases described worldwide, including Italy, are raising (Venco et al. 2011, Demiaszkiewicz et al. 2013, Ravindran et al. 2016, Omeragić et al. 2018, Napoli et al. 2019). In humans, scrotum and testicles seem to be common localizations for *D. repens*, probably due to lower body temperature of these areas and a tropism of *D. repens* to higher concentrations of sexual hormones. Furthermore, higher awareness of patients for these body parts could increase the detection of the parasite in these areas (Pampiglione and Rivasi 2000). The first two hypotheses could be plausible for dogs as well. In veterinary medicine, neutering young male dogs is common and recommended by practitioners. Frequent use of this practice in most of the canine population could explain why the localization in the genitalia seems to be less common in dogs then in humans. Indeed, the majority of reports on the testicular localization of *D. repens* are referring to kennel dogs or to dogs living in countries where systematic neutering is less practiced. The first testicular localization of *D. repens* in dogs was reported in Poland by Demiaszkiewicz et al. (2013). In their report, the dog’s testicle was enlarged and the parasite was found within a cystic-vascular Leydig cell tumor in the testicular parenchyma. In India, *D. repens* was found protruding from the *tunica vaginalis* incised during the neutering procedure of healthy dogs. The presence of the parasite was associated with thickening and enlarging of epididymis and testicles. Indeed, histopathological examination of testicles and epididymis revealed interductal fibrosis, venous congestion and widening of the cavernous spaces (Ravindran et al. 2016). Moreover, in Bosnia-Erzegovina, the parasite was found free or within cysts in the testicular subcutaneous connective tissue of a dog showing subcutaneous masses and nodules both on testicles and abdominal wall (Omeragić et al. 2018). In Italy, the parasite was observed in the vaginal cavity and within the parietal layer of *tunica vaginalis* without any phlogistic or degenerative changes of *tunica vaginalis*, testicle, and epididymis (Napoli et al. 2019). In this study, a *D. repens* gravid female was found on the surface of the parietal layer of *tunica vaginalis* of dog 1, with *tunica vaginalis*, testicle, and epididymis normal in shape and size, and without any gross lesions. The dog showed marked circulating eosinophilia (3.3·10^3^/μL) and no clinical signs. Hematological parameters are rarely evaluated in other studies focused on testicular localizations of *D. repens.* In a study by Napoli et al. (2019), an increase of the white blood cell count was found, but the differential white blood cell count was not assessed. In the study carried out by Omeragić et al. (2018), hematocrit and hemoglobin concentration were decreased, whereas white blood cells, neutrophils, and eosinophils (2.0·10^9^/L) were increased. In a case of subcutaneous localization of *D. repens* with concurrent microfilaremia by *D. immitis*, a moderate eosinophilia (2.7·10^9^/L) was observed (Giori et al. 2010). In human cases of dirofilariasis, eosinophilia is rarely observed (Joseph et al. 2011, Fontanelli-Sulekova et al. 2016, Ermakova et al. 2017, Kłudkowska et al. 2018). In a study on 266 patients with *D. repens* infection, peripheral blood eosinophilia was detected in 16.4% of cases and only in those patients showing migrating worms (Ermakova et al. 2017). Dirofilaria infection should be considered in the differential diagnosis of eosinophilia of unknown etiology (Kłudkowska et al. 2018). In dogs, parasitic infections, particularly by ectoparasites or parasite with a migrating tissue phase (e.g., *D. immitis*, *A. vasorum*), are the most common cause of eosinophilia. Other pathologies like allergies or hypersensitivity reactions, inflammatory bowel disease, paraneoplastic syndrome, Addison’s disease, eosinophilic diseases (myositis, gastroenteritis, pneumonia), idiopathic hypereosinophilic syndrome, and chronic eosinophilic leukemia can cause eosinophilia as well (Lilliehook et al. 2000, Lilliehook and Tvedten 2003, Mansfield 2008). Dog 1 was treated with broad-spectrum ecto- and endo-parasiticide drugs at kennel admission and was negative for lungworms by Baermann’s test. Furthermore, the dog did not show any symptom or change in hematochemical profile associated with other causes of eosinophilia; in addition, the number of eosinophils returned within the reference range after specific anti-filarial treatment. In the present case, *D. repens* could then be the cause of circulating eosinophilia. Dog 2 of this study had also microfilaremia and showed signs of pyotraumatic dermatitis. Also called acute moist dermatitis or “hot spot,” it is a rapidly developing bacterial skin infection secondary to self-inflicted trauma. However, the pathogenesis of pyotraumatic dermatitis is poorly understood. It has been speculated that local skin inflammation and pruritus could be the trigger for the development of these lesions: the repeated attempts to relieve the itching, through scratching, licking, or chewing can cause tissue damage and bacterial colonization (Cobb et al. 2005). Even though during *D. repens* infection pruritus was not always present, in a study involving 100 infected dogs, this symptom was observed in 100% of the cases and 6% showed eczema or pyoderma (Tarello 2011, Rocconi et al. 2012). Itching and dermatological manifestations could be induced by the movement of adult parasites in the subcutaneous tissues, immunological and allergic reactions to parasitic stages L3–L5 or microfilariae, capillary embolization of microfilariae, and toxins released in the circulatory system (Tarello 2002, Tarello 2011). In animals with microfilaremia and dermatological symptoms, cytology, histological examination, or skin ultrasounds are necessary to unequivocally correlate the lesions to the parasite (Giori et al. 2010; Venco et al. 2011; Albanese et al. 2013; Manzocchi et al. 2017). Without any evidence of *D. repens* within the skin lesion, it is not possible to exclude *a priori* its implication, since the parasite could act as a trigger and then migrate. In dog 2 of this study, no cytology, histological examination, or ultrasonography were performed to confirm the presence of larval stages or adult parasites within the lesion. Nevertheless, the implication of *D. repens* as trigger of the itch-scratch cycle cannot be excluded. In addition, the application of moxidectin 2.5%/imidacloprid 10%/kg was effective in treating dogs, as reported by other authors (Fok et al. 2010; Hellmann et al. 2011). After the first treatment and during three monthly follow-ups, both animals were negative for circulating microfilariae by modified Knott’s test and PCR. This status was maintained during the post-treatment observation period.

## Conclusion

*D. repens* has a zoonotic potential higher than *D. immitis* and its diffusion has been increasing in recent years (Simon et al. 2012, Genchi and Kramer 2017, Capelli et al. 2018). The classical localization of *D. repens* in dogs is in subcutaneous tissues, free or within nodules. However, the infection can also occur in testicles and be associated with circulating eosinophilia, as reported in this study. Aspecific dermatological symptoms including eczema and pyodermitis can be present with or without pruritus. Therefore, during the neutering procedures, it is advisable to carefully inspect the removed testicles and incising the *tunica vaginalis* in case of “closed technique,” since parasite localization can also take place without any symptoms or macroscopic changes. Also, in case of circulating eosinophilia or pyotraumatic dermatitis, investigations should include modified Knott’s test and PCR for *D. repens* to ensure that the parasite was not the cause of these alterations. In endemic areas, adequate chemoprophylaxis should be provided for all animals, while in non-endemic areas, it would be appropriate to test by modified Knott’s test and PCR all dogs coming from endemic countries. Finally, more efforts should be made to develop rapid and sensitive tests for the early detection of infected animals in order to limit and prevent the spread of this zoonosis.

## References

[CR1] Agapito D, Aziz N-AA, Wang T, Morgan ER, Wright I (2018). Subconjunctival Dirofilaria repens infection in a dog resident in the UK. J. Small Anim Pract.

[CR2] Albanese F, Abramo F, Braglia C, Caporali C, Venco L, Vercelli A, Ghibaudo G, Leone F, Carrani F, Giannelli A, Otranto D (2013). Nodular lesions due to infestation by *Dirofilaria repens* in dogs from Italy. Vet Dermatol.

[CR3] Capelli G, Genchi C, Baneth G, Bourdeau P, Brianti E, Cardoso L, Danesi P, Fuehrer HP, Giannelli A, Ionică AM, Maia C, Modrý D, Montarsi F, Krücken J, Papadopoulos E, Petrić D, Pfeffer M, Savić S, Otranto D, Poppert S, Silaghi C (2018). Recent advances on *Dirofilaria repens* in dogs and humans in Europe. Parasit Vectors.

[CR4] Casiraghi M, Anderson TJC, Bandi C, Bazzocchi C, Genchi C (2001). A phylogenetic analysis of filarial nematodes: comparison with the phylogeny of Wolbachia endosymbionts. Parasitology.

[CR5] Cobb MA, Edwards HJ, Jagger TD, Marshall J, Bowker KE (2005). Topical fusidic acid/betamethasone-containing gel compared to systemic therapy in the treatment of canine acute moist dermatitis. Vet J.

[CR6] Demiaszkiewicz AW, Karamon J, Jasik A (2013). Case of *Dirofilaria repens* in a testis of a dog. Med Weter.

[CR7] Ermakova L, Nagorny S, Pshenichnaya N, Ambalov Y, Boltachiev K (2017). Clinical and laboratory features of human dirofilariasis in Russia. IDCases.

[CR8] Fok E, Jacsó O, Szebeni Z, Győrffy A, Sükösd L, Lukács Z, Schaper R (2010). Elimination of *Dirofilaria* (syn. *Nochtiella*) *repens* microfilariae in dogs with monthly treatments of moxidectin 2.5%/imidacloprid 10% (Advocate®, Bayer) spot-on. Parasitol Res.

[CR9] Fontanelli Sulekova L, Gabrielli S, De Angelis M, Milardi GL, Magnani C, DiMarco B, Taliani G, Cancrini G (2016). *Dirofilaria repens* microfilariae from a humannode fine-needle aspirate: a case report. BMC Infect Dis.

[CR10] Genchi C, Kramer L (2017). Subcutaneous dirofilariosis (*Dirofilaria repens*): an infection spreading throughout the old world. Parasites Vectors.

[CR11] Giori L, Garbagnoli V, Venco L, Genchi M, Bazzocchi C, Bertazzolo W (2010). What is your diagnosis? Fine-needle aspirate from a subcutaneous mass in a dog. Vet Clin Pathol.

[CR12] Giudice E, Di Pietro S, Gaglio G, Di Giacomo L, Bazzano M, Mazzullo G (2014). Adult of *Dirofilaria repens* in a dog with recurrent multiplesubcutaneous nodular lesions. Parasitol Res.

[CR13] Hellmann K, Heine J, Braun G, Paran-Dobesova R, Svobodova V (2011). Evaluation of the therapeutic and preventive efficacy of 2.5% moxidectin/10% imidacloprid (Advocate®, Bayer Animal Health) in dogs naturally infected or at risk of natural infection by *Dirofilaria repens*. Parasitol Res.

[CR14] Hermosilla C, Pantchev N, Dyachenko V, Gutmann M, Bauer C (2006). First autochthonous case of canine ocular *Dirofilaria repens* infection in Germany. Vet Rec.

[CR15] Joseph E, Matthai A, Abraham LK, Thomas S (2011). Subcutaneous human dirofilariasis. J Parasit Dis.

[CR16] Kłudkowska M, Pielok Ł, Frąckowiak K, Masny A, Gołąb E, Paul M (2018). *Dirofilaria repens* infection as a cause of intensive peripheral microfilariemia in a Polish patient: process description and cases review. Acta Parasitol.

[CR17] Lilliehook I, Tvedten H (2003). Investigation of hypereosinophilia and potential treatments. Vet Clin Small Anim.

[CR18] Lilliehook I, Gunnarsson L, Zakrlssont G, Tvedten H (2000). Diseases associated with pronounced eosinophilia: a study of 105 dogs in Sweden. J Small Anim Pract.

[CR19] Manfredi MT, Vieira C., Bandi C., Casuraghi M., Simón F. (2001) Chapter I. Phylogeny, systematics and structural aspects. In: Heartworm infection in humans and animals. F. Simón and C. Genchi Editors, Ediciones Universidad de Salamanca, pp. 19–40

[CR20] Mansfield C (2008) Eosinophilic diseases of dogs. World Small Animal Veterinary Association World Congress Proceedings

[CR21] Manzocchi S, Lendner M, Piseddu E, Sebastiani V, Morabito S, Daugschies A, Pantchev N (2017). Nodular presentation of *Dirofilaria repens* infection in a cat mimicking a fibrosarcoma. Vet Clin Pathol.

[CR22] Mircean M, Ionică AM, Mircean V, Györke A, Codea AR, Tăbăran FA, Taulescu M, Dumitrache MO (2017). Clinical and pathological effects of *Dirofilaria repens* and *Dirofilaria immitis* in a dog with a natural co-infection. Parasitol Int.

[CR23] Omeragić J, Beck R, Klarić D, Bačić E (2018). *Dirofilaria repens* in canine testicles in Bosnia and Herzegovina. Veterinaria.

[CR24] Pampiglione S, Rivasi F (2000). Human dirofilariasis due to *Dirofilaria (Nochtiella) repens*: an update of world literature from 1995 to 2000. Parassitologia.

[CR25] Ravindran R, Julie B, Swapna SA, Jerin F, Jyothimol G, Lenka DR, Nandakumar S, Sabu SM (2016). *Dirofilaria repens* in scrotum of dogs. Trop Biomed.

[CR26] Rawlings CA (1986) Diagnosis of infection. pp. 209–229 in Pedersen, D. (Ed.) Heartworm disease in dogs and cats. Philadelphia, W.B. Saunders

[CR27] Rocconi F, Di Tommaso M, Traversa D, Palmieri C, Pampurini F, Boari A (2012). Allergic dermatitis by *Dirofilaria repens* in a dog: clinical picture and treatment. Parasitol Res.

[CR28] Simón F, Siles-Lucas M, Morchón R, González-Miguel J, Mellado I, Carretón E, Montoya-Alonso JA (2012). Human and animal dirofilariasis: the emergence of a zoonotic mosaic. Clin Microbiol Rev.

[CR29] Venco L, Valenti V, Bertazzolo W, Genchi M, Grandi G (2011). A miniinvasive procedure for removal of adult *Dirofilaria repens* from subcutaneous nodules in dogs. Int J Appl Res Vet Med.

